# Inclusion of a specific T cell epitope increases the protection conferred against foot-and-mouth disease virus in pigs by a linear peptide containing an immunodominant B cell site

**DOI:** 10.1186/1743-422X-9-66

**Published:** 2012-03-14

**Authors:** Carolina Cubillos, Beatriz G de la Torre, Juan Bárcena, David Andreu, Francisco Sobrino, Esther Blanco

**Affiliations:** 1 Centro de Investigación en Sanidad Animal (CISA-INIA), Carretera de Algete a El Casar, Valdeolmos, 28130 Madrid, Spain; 2 Departament de Ciències Experimentals i de la Salut, Universitat Pompeu-Fabra, 08003 Barcelona, Spain; 3 Centro de Biología Molecular "Severo Ochoa" (CSIC-UAM), Cantoblanco, 28049 Madrid, Spain

**Keywords:** Foot-and-mouth disease virus, FMDV, Linear peptides, Vaccine, Pig, Swine

## Abstract

**Background:**

Foot-and-mouth disease virus (FMDV) causes an economically important and highly contagious disease of cloven-hoofed animals. FMD control in endemic regions is implemented using chemically inactivated whole-virus vaccines. Currently, efforts are directed to the development of safe and marked vaccines. We have previously reported solid protection against FMDV conferred by branched structures (dendrimeric peptides) harbouring virus-specific B and T-cell epitopes. In order to gain insights into the factors determining a protective immune response against FMDV, in this report we sought to dissect the immunogenicity conferred by different peptide-based immunogens. Thus, we have assessed the immune response and protection elicited in pigs by linear peptides harbouring the same FMDV B-cell or B and T-cell epitopes (B and TB peptides, respectively).

**Results:**

Pigs were twice immunized with either the B-cell epitope (site A) peptide or with TB, a peptide where the B-cell epitope was in tandem with the T-cell epitope [3A (21-35)]. Both, B and TB peptides were able to induce specific humoral (including neutralizing antibodies) and cellular immune responses against FMDV, but did not afford full protection in pigs. The data obtained showed that the T-cell epitope used is capable to induce efficient T-cell priming that contributes to improve the protection against FMDV. However, the IgA titres and IFNγ release elicited by these linear peptides were lower than those detected previously with the dendrimeric peptides.

**Conclusions:**

We conclude that the incorporation of a FMDV specific T-cell epitope in the peptide formulation allows a significant reduction in virus excretion and clinical score after challenge. However, the linear TB peptide did not afford full protection in challenged pigs, as that previously reported using the dendrimeric construction indicating that, besides the inclusion of an adecuate T-cell epitope in the formulation, an efficient presentation of the B-cell epitope is crucial to elicit full protection by peptide vaccines.

## Background

Foot-and-mouth disease (FMD) is a highly infectious disease of cloven-hoofed animals, and probably the most important livestock disease in terms of economic impact [1-3]. In many areas of the world (Africa, Asia and to some extent, South America) FMD remains endemic causing severe handicap for access to international markets [4]. This endemic situation poses a constant threat to countries that have a FMD-free status, which has been increased over the last decade by the accelerated trade and movements of people due to globalization [5]. The risk of FMD introduction and spread into countries or zones declared officially free has been confirmed by FMD outbreaks such as those in United Kingdom and the Netherlands (2001), China (2005), Russia, Brazil and Argentina (2006) [6], and more recently in Japan, Republic of Korea, China and Mongolia (2010) (OIE information Database).

FMD control in endemic regions is mainly implemented by using chemically inactivated whole-virus vaccines. The basic technology for vaccine production, which has remained the same for decades, requires the growth of large volumes of virulent FMDV, subsequent virus inactivation, antigen concentration and purification [2,7]. This raises concerns on biosafety issues, as the risk of virus release during vaccine production [2,8]. Additional shortcomings of current FMD vaccines include: i) lack of long-term protection, making multiple vaccinations necessary; ii) Thermal instability, requiring an adequate cold chain); iii) vaccinated animals exposed to infection can become asymptomatic carriers, and iv) depending upon the manufacturer, vaccines can contain traces of non-structural proteins (NSP) making it difficult to distinguish between vaccinated and infected animals when using currently approved assays [2,7,9]. These concerns along with the severe trade restrictions applied in case of any vaccination campaign, have led FMDV-free countries to adopt a non-vaccination policy that relies on slaughtering infected and contact herds, and the strict limitations on animal movements [10]. Therefore, much effort has been invested in search of alternative, safe and marked vaccines. Based on the virus capsid structure and one main B-cell antigenic sites identified [11,12], a number of strategies to develop new, alternative FMD vaccines have been used. Among them, the use of synthetic peptides offers clear advantages over conventional vaccines addressing most of the above mentioned caveats. The relatively simple production of clinical grade, easily characterized vaccine peptides facilitates quality control and regulatory approval, in addition to allowing swift changes in design and thus rapid translation of new immunological concepts [13]. Even more significant is the fact that peptide-based vaccines are by nature free of any infectious component and thus inherently fulfill the requirement of allowing differentiation of vaccinated from infected animals (DIVA) [14].

Linear peptides spanning epitopes from VP1 of FMDV have provided limited protection to viral challenge in natural hosts [12,15]. The lack of T cell epitopes widely recognized by T cells from individuals of domestic populations of natural hosts, and capable of providing adequate co-operation to immune B lymphocytes, has been proposed as one of the limiting factors for the development of efficient FMD peptide vaccines [16]. Recently we have reported solid protection against FMDV challenge in pigs immunized with a dendrimeric peptide construct [17] consisting of one copy of a T-cell epitope [3A(21-35)] frequently recognized by outbred pigs [18] that branches out into four copies of a B-cell epitope (site A). This dendrimeric construction specifically induced high titers of FMDV-neutralizing antibodies, the activation of T-cells, release of IFNγ and a potent anti-FMDV IgA responses (systemic and mucosal) [17].

To better understand the determinants of the protection conferred by this dendrimeric peptide, we have assessed the immune response and protection elicited in pigs by linear peptides containing the same B- and T-cell FMDV epitopes displayed in the dendrimeric peptide. Two groups of pigs were immunized with either the B peptide or with TB, a peptide in which the B-epitope was in tandem with the T-cell epitope (Table 1). Both linear peptides were able to induce specific humoral (including neutralizing antibodies) and cellular immune response to FMDV, and conferred partial protection upon viral challenge, characterized by a delay on the onset of signs which were less severe than those observed in control non-immunized pigs. Interestingly in the peptide-immunized animals, a post-challenge reduction of FMDV excretion, more significant in TB peptide-immunized pigs, was found. Compared with the immune response elicited by the dendrimeric peptide reported before [17], the more significant differences found for the present set of peptides concerned IgA titres and IFNγ release, both displaying much lower levels than those achieved with the dendrimeric peptide, indicating a potential role of both effector mechanisms in the protection against FMDV induced by such peptide.

**Table 1:** Synthetic peptides used in this study

Peptide	FMDV protein (residues)	Sequence
**B**	VP1 [136-154]	YTASARGDLAHLTTTHARH- amide
**T**	3A [21-35]	AAIEFFEGMVHDSIK- amide
**TB**	3A [21-35] VP1 [136-154]	AAIEFFEGMVHDSIKYTASARGDLAHLTTTHARH- amide

## Results

### Immunization with peptides B and TB affords partial clinical protection to FMD challenge

**Figure 1:**
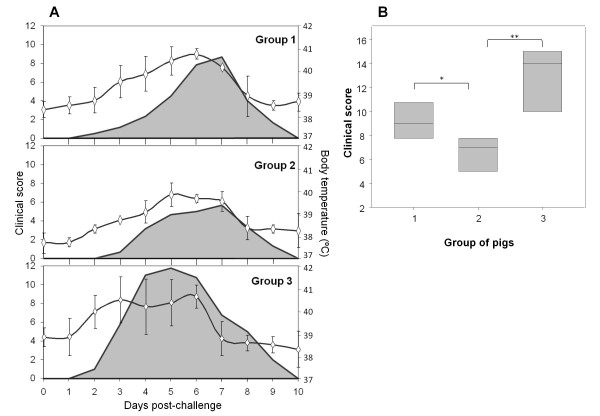
**A) Time course of clinical disease in challenged pigs immunized with peptide B (group 1), peptide TB (group2), and in non-immunized pigs (group 3), after challenge with FMDV C-S8c1**. The mean body temperature (°C) [right, rhombs] and the score of the clinical signs (grey curve; calculated as indicated in Materials and Methods) are shown. **B) **Box plot showing the range of the maximum clinical scores (see Materials and Methods) recorded for the individual animals from groups 1, 2 and 3 after challenge. Statistically significant differences were found in the median values (line into the box) between groups 2 and 1 (* *P *= 0.026) and 2 and 3 (** *P *= 0.003).

Domestic Landrace x Large White pigs distributed in three different groups, were vaccinated twice with B peptide (group 1), TB peptide (group 2) or PBS and challenged with type C FMDV (isolate C-S8c1). Animals were examined daily monitoring rectal temperatures, and a protection score (see details in Material and Methods). Body temperatures above 39.5°C were detected in pigs from group 3 (infection control group) at 2 days post-challenge (Figure 1A). Two out of four pigs had elevated temperatures above 41°C for more than two days. In these animals, small vesicular lesions were found on the snout, lips and tongue, as well as regular vesicles in the interdigital area or along coronary band of more than two feet, around 3 days post-challenge. At four days post-challenge, three out of four pigs from group 3 developed generalized clinical signs (scored as 11,14 and 15) while the fourth showed clear FMDV signs (score of 4), and the mean clinical score of the group was 11 (Figure 1A).

None of the twelve pigs immunized with B or TB peptides developed temperatures above 41°C during the 10 days post-challenge monitored (Figure 1A). In these pigs a delay of two days in the detection of pyrexia (from day 4 post-challenge) relative to control challenged animals was noticed. In addition, the presence of vesicular lesions in more than two feet was found delayed, around 5 and 6 days post-challenge in groups 1 and 2, respectively. Likewise, fifty per cent of the animals immunized with peptide B (pigs B1, B2 and B5) and only one of the six pigs immunized with TB peptide (pig TB6) showed generalized FMD, which appeared significantly delayed (7 days post-challenge), compared to those developed by control pigs (group 3). The maximum clinical scores (mean) registered along the post-challenge time course were 9.8 (group 1), 6.8 (group 2) and 13,7 (group 3) (Figure 1B). These differences were statistically significant (*P *< 0.05) between group 2 (peptide TB) and groups 1 and 3 (peptide B and control infection group, respectively), suggesting that the presence of the T cell epitope enhances the protection conferred.

### Reduction of virus excretion after challenge in peptide-vaccinated pigs

The time course of virus shedding in the challenged pigs was analyzed by means of viral RNA detection in samples collected at days 3, 7 and 10 post-infection (Table 2). In group 3 (infection control animals) the four pigs were positive for FMDV RNA in blood (viremia), nasal and pharyngeal samples at 3 days post-infection. Blood and pharyngeal swabs from two control pigs remained positive at 7 days post-infection. Virus RNA could be detected in nasal swabs from the four animals for the entire period of examination, except for pig C4 at 10 days post-infection. On the other hand, the total number of samples positive for viral RNA in groups 1 and 2 (peptide-immunized pigs) was clearly lower (18 and 4 out of 42 samples tested, respectively) compared to those detected in the group 3 (23 out of 36 samples tested). At 7 days post-infection the viral RNA detection pattern in both immunized groups was clearly different; while in group 1 (B peptide-immunized animals) swab samples from three pigs were positive, in group 2 (TB peptide-immunized animals) only one positive sample was detected. At 10 days post-infection all the samples tested from both groups (1 and 2) were FMDV RNA negative.

**Table 2:** Detection of FMDV RNA in blood and respiratory tract samples (nasal and pharyngeal swabs) from challenged pigs

			Day post-challenge											
			**0**			**3**			**7**			**10**		

**Group**	**Inoculum**	**Pig**	**B^a^**	**N^b^**	**P^c^**	**B**	**N**	**P**	**B**	**N**	**P**	**B**	**N**	**P**
1	B peptide	**B1**	**-**	na^d^	na	**+**	na	na	**-**	na	na	**-**	na	na
		**B2**	**-**	na	na	**+**	na	na	**+**	na	na	**-**	na	na
		**B3**	**-**	**- **^e^	**-**	**-**	**+ **^f^	**+**	**-**	**-**	**-**	**-**	**-**	**-**
		**B4**	**-**	**-**	**-**	**-**	**-**	**+**	**-**	**+**	**+**	**-**	**-**	**-**
		**B5**	**-**	**-**	**-**	**+**	**-**	**+**	**+**	**+**	**+**	**-**	**-**	**-**
		**B6**	**-**	**-**	**-**	**+**	**+**	**+**	**-**	**+**	**+**	**-**	**-**	**-**

2	TB peptide	**TB1**	**-**	na	na	**+**	na	na	**-**	na	na	**-**	na	na
		**TB2**	**-**	na	na	**-**	na	na	**-**	na	na	**-**	na	na
		**TB3**	**-**	**-**	**-**	**+**	**-**	**+**	**-**	**-**	**-**	**-**	**-**	**-**
		**TB4**	**-**	**-**	**-**	**-**	**-**	**+**	**-**	**-**	**-**	**-**	**-**	**-**
		**TB5**	**-**	**-**	**-**	**-**	**-**	**-**	**-**	**-**	**-**	**-**	**-**	**-**
		**TB6**	**-**	**-**	**-**	**-**	**-**	**-**	**-**	**-**	**+**	**-**	**-**	**-**

3	PBS	**C1**	**-**	-	-	**+**	**+**	**+**	**+**	**+**	**+**	**-**	**+**	-
		**C2**	**-**	-	-	**+**	**+**	**+**	**-**	**+**	-	**-**	**+**	-
		**C3**	**-**	-	-	**+**	**+**	**+**	**-**	**+**	-	**-**	**+**	-
		**C4**	**-**	-	-	**+**	**+**	**+**	**+**	**+**	**+**	**-**	-	-

^a ^Blood

^b ^Nasal swabs

^c ^Pharyngeal swabs

^d ^sample not available

^e ^RNA not detected

^f ^RNA detected

To further determine the relative level of FMDV replication in the different pig groups, at 10 days post-infection during the necropsy, several tissues were collected and tested for FMDV RNA detection (Table 3). Similar to viral RNA detection in blood and swabs, the number of RNA positive tissues was lower in group 2 compared to groups 1 and 3, excluding lymph nodes that were positive in all the pigs. Overall, as found with the severity of the clinical signs, a lower extent of FMDV replication was observed in challenged animals immunized with peptide TB.

**Table 3:** Detection of FMDV RNA in tissue samples collected post-mortem (10 days post-challenge)

Group	Inoculum	Pig	Detection of FMDV RNA						
			**Heart**	**Lung**	**Spleen**	**Liver**	**Kidney**	**Tonsil**	**Lymph node**

1	B peptide	**B1**	-	-	+	-	-	-	+
		**B2**	+	-	+	+	-	+	+
		**B3**	-	-	-	-	-	-	+
		**B4**	-	-	-	-	-	+	+
		**B5**	+	-	+	+	-	+	+
		**B6**	-	-	+	-	-	+	+

**2**	TB peptide	**TB1**	**-**	**-**	**-**	**-**	**-**	**-**	**+**
		**TB2**	-	-	-	-	-	-	+
		**TB3**	+	-	-	-	-	-	+
		**TB4**	-	-	-	-	-	+	+
		**TB5**	-	-	-	+	-	-	+
		**TB6**	-	-	+	-	-	+	+

3	PBS	**C1**	+	-	+	+	+	+	+
		**C2**	+	-	+	-	+	+	+
		**C3**	-	-	+	-	+	+	+
		**C4**	-	-	+	+	-	+	+

### Peptides B and TB elicit neutralizing and specific IgG1, IgG2 and IgA antibodies

Upon revaccination, all the 12 immunized pigs (groups 1 and 2) showed a rise in serum neutralizing antibody titres at day 39 (18 days post-boost), which increased significantly (*P *< 0.05) again at day 49 (10 days after challenge) (Table 4). No significant VN-titre differences were detected between groups 1 (B-immunized pigs) and 2 (TB-immunized pigs) at day 39, and after FMDV challenge at day 49. In these animals, no rise of VN-titres was detected after a single peptide dose (day 21), except in two animals: B1 (group 1) and TB5 (group 2) (Table 4).

**Table 4:** Serum neutralising antibodies and isotype-specific responses (IgG1, IgG2 and IgA) in serum samples from challenged pigs

			VNT		IgG1		IgG2		IgA	
**Group**	**Inoculum**	**Pig**	**39^a^**	**49^b^**	**39^a^**	**49^b^**	**39**	**49**	**39**	**49**

1	B peptide	**B1**	2.5	2.8	5,0	4,1	4,4	4,3	4,1	3,7
		**B2**	2,2	2.5	2,7	3,0	2,5	2,7	3,0	2,8
		**B3**	2.5	3.4	3,4	2,8	3,1	2,7	2,8	3,0
		**B4**	2.2	3.4	2,7	2,9	2,4	3,0	2,4	2,8
		**B5**	1.9	3,7	2,3	3,6	2,2	3,8	2,7	3,0
		**B6**	2,2	3,1	2,5	3,1	2,1	3,0	2,2	3,0
		**mean**^c^	2,2	3,1	3,0	3,2	2,7	3,2	2,8	3,0
		**sd**^d^	± 0.2	± 0.4	± 1	± 1	± 1	± 1	± 0,7	± 0,3

2	TB peptide	**TB1**	2,5	2,8	2,0	2,1	2,0	2,0	2,3	2,5
		**TB2**	2,2	2,8	2,8	2,8	2,6	2,6	2,7	2,8
		**TB3**	1.9	2,5	2,3	2,8	2,2	2,7	2,1	2,7
		**TB4**	1.9	3.1	2,0	2,8	2,4	3,2	2,5	2,7
		**TB5**	3.1	3,4	2,6	3,1	2,6	3,3	2,6	2,9
		**TB6**	2.5	2.8	3,2	3,6	2,9	3,4	2,9	3,0
		**mean**	2,3	2,9	2,4	2,8	2,4	2,8	2,5	2,8
		**sd**	± 0,4	± 0,3	± 0,5	± 0,5	± 0,3	± 0,5	± 0,3	± 0,2

3	PBS	**C1**	-	3.1	-	3	-	2,9	-	3,5
		**C2**	-	2,8	-	2,5	-	2,2	-	2,7
		**C3**	-	3.1	-	2,6	-	2,3	-	2,9
		**C4**	-	2,8	-	2,0	-	2,4	-	2,7
		**mean**		2,9		2,5		2,4		2,9
		**sd**		± 0,1		± 0,4		± 0,3		± 0,4

^a ^Sample collected at day 39 (18 days after peptide boost).

^b ^Sample collected at day 49 (10 days post-challenge).

^c ^Mean isotype titres.

^d ^Standard deviation.

^- ^Sample not available.

All immunized pigs showed detectable serum levels of IgG1, IgG2 and IgA antibodies against FMDV on day 39 (18 days post-boost) (Table 4), but not after the first immunization (data not shown). Since the isotype-specific assays used were not absolute quantitative (did not include known isotype standars), only comparisons between animal groups for individual isotypes were possible. No significant differences in IgG1, IgG2 and IgA titres were found between groups 1 and 2 at days 39 and 49, nor compared to the isotypes detected in group 3 (infection control pigs) at day 49.

Therefore, these results suggest that the presence of the T-cell epitope does not modify the magnitude or isotype switching of the antibody response. However, differences in antibody affinities induced by peptides B and TB, which might enhance the protection conferred, cannot be ruled out.

### Peptides B and TB induce FMDV-specific T cell responses

**Figure 2:**
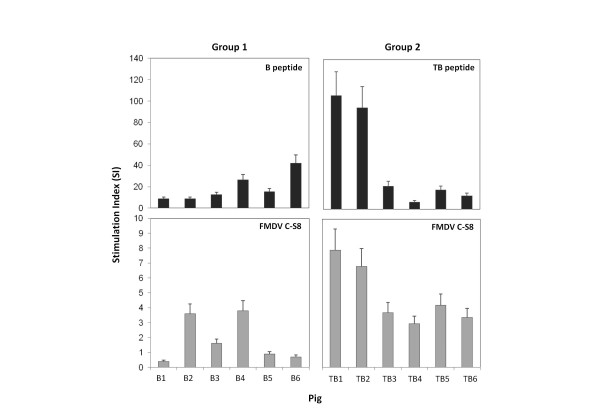
**Proliferative response against peptides (20 μg/ml per well) and virus (5·10^5 ^pfu/ml) of PBMCs obtained at day 39 from peptide-immunized pigs (group 1 and 2)**. Results are expressed as SI and each bar represents the mean of triplicate cultures. The background cpm values (obtained with lymphocytes incubated with medium alone or mock-infected cells) were always ≤ 2300 cpm.

Induction of FMDV-specific T-cells was analyzed by lymphoproliferation assays with PBMCs collected from peptide-immunized pigs at days 0, 21 and 39, and stimulated *in vitro *with peptides (B or TB) or virus. No stimulation in response to peptides or virus was observed with PBMCs obtained from the animals prior to immunization (day 0). After the first immunization, day 21, specific responses to peptides started to be detectable with SI (< 4) and such responses were not observed for whole-virus antigen (data not shown). Higher specific responses (SI > 6) against peptides B or TB were found for lymphocytes from all peptide-immunized pigs at day 39 (18 days post-boost) (Figure 2). The magnitude of these responses showed animal-to-animal variation, but a consistent trend was noticed: responses against peptide and virus were higher in TB-immunized pigs than in B-immunized pigs. In all cases, maximal responses were induced with 20 μg/ml of peptides B or TB.

The lymphoproliferative response against whole-virus antigen significantly differed between groups 1 and 2. All animals immunized with peptide TB (group 2) responded to virus with SI > 2.5. However, PBMCs from only two of the six animals immunized with peptide B (group 1) significantly proliferated against virus stimulation (SI > 2) (Figure 2). In addition, a correlation was observed between response against peptide and whole-virus antigen in group 2 but not in group 1.

Production of IFN-γ and IL10 was analyzed in supernatants of PBMCs from immunized pigs at day 39, in response to *in vitro *stimulation with peptides B or TB, as well as in supernatants from mock-stimulated cultures. Low levels of IFNγ production (8-10 pg/ml), were detected in supernatants of PBMCs from pigs TB4 and TB5 (group 2), in response to *in vitro *stimulation with peptide TB but not in mock-stimulated cultures (data not shown). Production of this Th1 cytokine was not detected in any of the B peptide-immunized pigs (group 1). Likewise, no IL10 was detected in pigs from either of the groups (data not shown).

These results support the theory that the presence of the T-cell epitope favors the priming of specific T-cells in the immunized animals, which could be recalled efficiently upon FMDV stimulation.

## Discussion

Vaccines based on synthetic peptides offer several advantages when compared with conventional vaccines based on attenuated or inactivated microorganisms, particularly regarding safety, thermal stability and ease of production. Despite the potential advantages of this approach, the development of successful peptide vaccines has been limited mainly by difficulties associated with *in vivo *stability, poor immunogenicity of linear peptides, and by the MHC polymorphism of the host species [19,20]. However, recent advances on the requirements for induction and maintenance of immune responses, as well as on the pharmacokinetics of peptides, have provided new strategies to enhance both, peptide immunogenicity and stability, which are leading to the return of peptide based technologies to the forefront of vaccine design [21,22].

The use of one of such approach, the multimerization of peptides by dendrimeric constructs, has recently allowed us to report solid protection against FMDV challenge [17]. The peptide dendrimer used B_4_T, contained four copies of an immunodominant B-cell epitope, named site-A, branched out a selected Th epitope of FMDV broadly recognized by T-cells from outbred pigs [18]. This dendrimeric construction specifically induced high titers of FMDV-neutralizing antibodies, activated T-cells, release of IFNγ and a potent anti-FMDV IgA response [17]. However, whether the immunogenicity and protection elicited by this dendrimeric peptide was due to incorporation of a relevant T-cell epitope and/or to the presence of a dendrimeric structure containing repeated B-cell epitopes remained to be addressed.

As part of the understanding of the determinants of the protection afforded by peptide B_4_T, we have analyzed the immune response and the protection conferred in pigs by linear peptides containing the B- and the T-cell epitopes displayed in the dendrimeric peptide.

As shown in Figure 1, immunization of pigs with linear peptides (B or TB) was not able to confer full protection as that reached using peptide B_4_T. However, significant differences on the maximum clinical scores were observed between the two groups of immunized animals (Figure 1B), being the values lower for animals immunized with peptide TB (group 2) (Figure 1B). These results correlate with the significant reduction of FMDV RNA detection after viral challenge in blood, nasal and pharingeal swabs in animals immunized with peptide TB (Table 2). Therefore, vaccination with a linear peptide displaying a specific T-cell epitope, can reduce virus excretion in pigs, which might contribute to reduced transmission of FMDV in the field, even if the pigs are not fully protected. Similar improved control of FMDV replication in mice has been achieved by DNA vaccines encoding one B and two T cell epitopes [23]. However, to reach the same protection results in pigs, the B and T epitopes encoded by the DNA vaccine requires to be tagerted to Class-II swine leukocyte antigens [24].

The protection against FMDV conferred by conventional whole virus vaccine broadly correlates with the virus neutralizing antibody levels present in serum in cattle and, to a lesser extent, in pigs [7,25,26]. Our results show a lack of correlation between neutralizing antibody levels and protection, as the levels of virus neutralizing antibodies induced by both peptides B and TB were similar (around 2 log10), and even similar to those previously reported for peptide B_4_T [17]. A similar lack of correlation has been reported in animals immunized with vaccines different from those based on inactivated FMDV [27].

Antibodies may neutralize FMDV by different mechanisms [28]. Thus, the lower protective effect of the antibodies induced by linear peptides (B or TB) could be due to differences in either the mechanisms of neutralization they exert (i.e. direct neutralization of receptor binding, Fc-γ-receptor-dependent viral clearance, complement-mediated lysis, antibody-dependent cytotoxicity) or to affinity differences. To assess other humoral factors potentially contributing to limited protection conferred by the linear peptides, the spectrum of immunoglobulin subclasses induced by peptides T and TB was analysed. Our results show that linear peptides were able to induce significant titres of specific IgG1, IgG2 and IgA at day 39 (18 days after boost). The magnitude of these isotype responses was similar after B and TB immunization, suggesting that the inclusion of the T-cell epitope has no influence on the antibody isotype switch. However, the IgA titres induced by both linear peptides are between 1 and 2 orders of magnitude lower than those reported previously for dendrimer vaccination [17]. Therefore, IgA induction seems to be dependent on the branched structure and not only on the presence of the T-cell epitope. Induction of IgA responses has been recently correlated with complete protection against FMDV challenge of pigs immunized with a highly concentrated, inactivated vaccine [29]. All together, this result indicates that the lack of full protection conferred by linear peptides is, a large extend due to the inefficient induction of specific IgA.

The highly repetitive array of epitopes on viral surfaces allows efficient crosslinking of antigen-specific immunoglobulins on B cells, constituting a strong activation signal that may even overcome B cell tolerance [30]. In contrast to linear monomeric peptides, dendrimers display a repetitive configuration, which could allow direct activation of B cells, leading to a rapid B cell proliferation and production of antibodies. Likewise the triggered B cells are able to activate T helper cells, leading to cytokine secretion that can result in a suitable environment for generation of B cell memory. Therefore the activation of these early mechanisms by dendrimeric peptides, but not by monomeric peptides, can be relevant for the induction of protective immune responses against FMDV. Further studies are required to test this hypothesis, by analyzing the interaction between dendrimeric versus monomeric peptides with different antigen presenting cells essential in the early specific responses (B cells, Dendritic cells, γδ T lymphocytes, etc.).

Besides the direct neutralization of viral infectivity, other mechanisms of viral clearance may operate *in vivo*, as T-cell activation and the balance of cytokines they release. Immunization with peptides B or TB elicited T cells that consistently proliferate when stimulated with the peptides (Figure 2). However, the lymphoproliferative response to FMDV was significantly higher for TB-immunized pigs (Figure 2). These results show that the inclusion of the specific T-cell epitope in the peptide formulation allows priming of T cells that can recognize more efficiently the viral epitopes presented in the context of a subsequent virus encounter.

Upon *in vitro *stimulation, primed T cells from two pigs immunized with the linear peptide TB released IFNγ, which was not detected in any of the animals immunized with peptide B. These results indicate that the inclusion of the FMDV T-cell epitope enhances the IFNγ release. However, since the IFNγ release is higher after dendrimer vaccination [17], we can assume that optimal IFNγ production requires the inclusion of an efficient T-cell epitope, as well as a suitable configuration. IFNγ is a major activator of macrophages, enhancing their antimicrobial activity and their capacity for processing and presenting antigens to T lymphocytes [31]. It has been reported that IFNγ stimulates MHC expression in antigen-presenting cells and that efficiently inhibits FMDV replication [32]. Therefore, these results suggest that the better clinical protection conferred by the TB peptide compared to B peptide, is mostly due to the induction of a more efficient lymphoproliferative response and IFNγ release.

Taken together, our results show that the incorporation of a specific T-cell epitope in the peptide formulation seems to be necessary but not sufficient to enhance the protective cellular response to the protective levels achieved by a dendrimeric peptide. Thus, even though further work is required to understand the details of mechanisms leading to the solid protection conferred by dendrimeric peptides, our results suggest that branched configuration along with the inclusion of a T-cell epitope are essential to induce protective immune responses characterized mainly by high specific IgA responses and production of IFNγ.

## Conclusions

From the results obtained in this study, we conclude that the incorporation of a FMDV specific T-cell epitope in the peptide formulation allows a significant reduction in virus excretion and clinical score after challenge. However, the linear TB peptide did not afford full protection in challenged pigs, as that previously reported using the dendrimeric construction indicating that, besides the inclusion of an adequate T-cell epitope in the formulation, an efficient presentation of the B-cell epitope is crucial to elicit full protection by peptide vaccines.

## Methods

### Synthetic peptides

Peptides in Table 1 were prepared by automated synthesis performed in an Applied Biosystems model 433 syntesizer employing standard Fmoc chemistry and 0.1 mmol FastMoc protocols on Fmoc-Rink-amide-MBHA resin, with 10-fold excess of Fmoc-protected-L-amino acids and HBTU coupling chemistry. Protected peptide resins were N-deblocked prior to full deprotection and cleavage with trifluoroacetic acid-water-triisopropylsilane (95:2.5:2.5 v/v, 90 min, rt). Peptides were precipitated by addition of chilled diethyl ether, taken up in aqueous HOAc (10% v/v) and lyophilized. Analytical reversed-phase HPLC was performed on a C_18 _column (4.6 × 50 mm, 3 μm), solvent A 0.045% TFA in H_2_O, solvent B 0.036% in ACN, flow rate 1 ml/min, UV detection at 220 nm. Preparative HPLC was performed on C_18 _column (21.2 × 250 mm, 10 μm), solvent A 0.045% TFA in H_2_O, solvent B 0.036% in ACN, flow rate 25 ml/min. Linear gradients of solvent B into A were used for elution. Fractions of adequate purity (HPLC > 95%) and with the expected mass (MALDI-TOF, Voyager DE-STR, Applied Biosystems, Foster City, CA, α-hydroxycinnamic acid matrix; spectra obtained in the reflector mode) were combined and lyophilized.

### Virus

A virus stock derived from type C FMDV isolate C-S8c1 (18) by two passages in BHK-21 cells, which maintained the consensus sequences at the capsid protein region [33], was used in this study.

### Immunization and infection of pigs

Two vaccine-challenge experiments were carried out involving 16 domestic Landrace x Large White 8-weeks-old pigs, distributed in three different groups. Two of these groups, consisting of 6 pigs each, were vaccinated twice by intramuscular injection with 1 ml of 0.35 μM B peptide (group 1) or TB peptide (group 2), emulsified with complete Freund's adjuvant at day 0, and with incomplete Freund's adjuvant at day 21. The third group of 4 pigs (group 3) was used as infection-control group. Pigs were housed in a high-containment unit, each group in a separate room. Eighteen days after boost, animals were challenged intradermally in the left rear foot with 10^4 ^PFU of FMDV C-S8c1. Animals were examined daily monitoring rectal temperatures, and a protection score based on the time of appearance and the number and severity of lesions was determined. Total protection was defined as complete absence of lesions (score 0) and score values below 8 were considered as partial protection Clinical score was calculated as follows [34]: i) an elevated body temperature of 40°C (score of 1), > 40.5 (score of 2), or > 41 (score 3); ii) reduced appetite (1 point) or no food intake and food left over from the day before (2 points); iii) lameness (1 point) or reluctance to stand (2 points); iv) presence of heat and pain after palpation of the coronary bands (1 point) or not standing on the affected foot (2 points); v) vesicles on the feet, dependent on the number of feet affected, with a maximum of 4 points; and vi) visible mouth lesions on the tongue (1 points), gums or lips (1 point) or snout (1 point), with a maximum of 3 points.

All experiments with live animals were performed under the guidelines of the European Union (EU directive 86/609/EEC) and were approved by the site ethical review committee.

### Virus neutralization test (VNT)

Virus-neutralizing activity was determined in sera using a standard micro-neutralization test performed in 96-well plates by incubating serial two-fold dilutions of each serum with 100 TCID50 (50% Tissue Culture Infective Dose) of FMDV C-S8c1 for 1 h at 37°C. Remaining viral activity was determined in 96-well plates containing fresh monolayers of BHK-21 cells. End-point titres were calculated as the reciprocal of the final serum dilution that neutralized 100 TCID50 of FMDV C-S8c1 in 50% of the wells [35].

### Detection of isotype-specific FMDV antibodies

FMDV-specific IgG1, IgG2 and IgA in sera were measured using monoclonal antibodies specific for these isotypes supplied by Serotec, and 100 μL of duplicate threefold dilution series of each serum (starting at 1/50), as described [17]. In these assays the point on the titration curve corresponding to A_492 _of 1.0 invariably fell on the linear part of the curve. Antibody titers were therefore expressed as the reciprocal of the last dilution calculated by interpolation to give an absorbance of 1.0 OD unit above background.

### Lymphoproliferation assays

Proliferation assays of swine lymphocytes were performed as described [17,18]. Blood was collected in 5 μM EDTA and used immediately for the preparation of peripheral blood mononuclear cells (PBMCs). Assays were performed in 96-well round-bottomed microtiter plates (Nunc). Briefly, 2.5 × 10^5 ^PBMCs per well were cultured in triplicate, in a final volume of 200 μL, in complete RPMI 10% (v/v) FCS, 50 μM 2-mercapto-ethanol, in the presence of various concentrations of: i) FMDV, ranging from 3 × 10^5 ^to 2 × 10^3 ^PFU, ii) synthetic peptides, ranging from 50 μg/mL to 10 μg/mL. Cultures with medium alone or with mock-infected cells were included as control for each blood sample. Cells were incubated at 37°C in 5% CO2 for 4 days. Following incubation, each well was pulsed with 0.5 μCi of methyl- [3H] thymidine for 18 h. Cells were collected using a cell harvester and the incorporation of radioactivity into the DNA was measured by liquid scintillation in a Microbeta counter (Pharmacia). Results were expressed as stimulation indexes (SI) that were calculated as the mean cpm of stimulated cultures/mean cpm of cultures grown in the presence of medium alone (peptide) or mock-infected cells (virus).

### Cytokine detection

PBMC supernatants were cultured with 20 μg/ml of B or TB peptides for 48 h and analyzed for cytokine expression using interleukin-10 (IL-10) CytoSets (Biosource) and gamma interferon (IFNγ) ELISA (Pierce, Endogen) kits. In each assay the corresponding recombinant porcine cytokine was diluted over the detection range recommended by the manufacturer to generate a standard curve from which sample concentrations (in pg/ml) were calculated.

### RT-PCR

FMDV RNA in blood and in nasal and pharyngeal swabs, as well as in different biological samples collected at sacrifice day, was amplified by RT-PCR and detected as described [36]. We used the Primers A (5'-ACACGGCGTTCACCCA(A/T)CGC-3') and B (5'-GACAAAGGTTTTGTTCTTGGTC-3') designed to bracket a 290-bp region in the FMDV 3D polymerase gene.

### Statistical analysis

The statistical significance of differences in the clinical score values and antibody isotype titers between groups were calculated by the Mann-Whitney rank sum test. The Student's *t *test was used for VNT comparisons between groups 1 (B-immunized pigs) and 2 (TB-immunized pigs). Statistical analyses were performed using the SigmaStat software.

## Competing interests

EB, JB, CC, FS, DA and BGT, hold a patent (P200602142) on the dendrimeric FMDV peptide cited on the manuscript.

## Authors' contributions

BGT and DA, generated the peptide contructions. CC and EB, carried out most of the experiments described in the manuscript; DA, FS and EB, conceived the study, participated in its design and coordination; JB, DA, FS and EB were involved in drafting the manuscript. All authors read and approved the final manuscript.
